# Rupture bilatérale des tendons rotuliens chez un sujet jeune sans notion de maladies systémiques ou de traitement par les corticostéroïdes: à propos d'un cas et revue de la literature

**DOI:** 10.11604/pamj.2014.19.49.5268

**Published:** 2014-09-22

**Authors:** Aniss Chagou, Abdelkarim Rhanim, Mohammed Ali Berrady, Moulay Omar Lamrani, Mohammed Oudghiri, Mohammed Saleh Berrada, Moradh El Yaacoubi

**Affiliations:** 1 Service de Traumatologie-Orthopédie, Centre Hospitalier Universitaire Avicenne, Rabat, Maroc, Université Mohammed V, Rabat, Maroc

**Keywords:** Rupture bilatérale, tendon rotulien, maladies systémiques, corticostéroïdes, Bilateral rupture, patellar tendons, systemic disease, corticosteroid therapy

## Abstract

Les lésions du tendon rotulien sont moins communes que celles du tendon quadricipital. Les lésions bilatérales sont encore plus rares et sont souvent associées à une notion de tendinopathie, d'injection de corticoïdes ou de maladies systémiques tels que le lupus érythémateux, l'ostéomalacie ou l'insuffisance rénale chronique. Nous rapportons le cas d'un patient de 26 ans victime d'une rupture bilatérale du tendon rotulien suite à une réception de saut. Le patient n'avait pas d'antécédents de tendinopathie ni de maladies systémiques. Le diagnostic a été suspecté devant une position anormalement haute des deux rotules avec une impossibilité d'extension active des deux jambes. L’échographie a confirmé le diagnostic. Le patient a été traité par la technique de laçage selon Judet protégée par un cadrage. La rupture bilatérale du tendon rotulien est rare. La plupart des patients rapportent une notion de maladie systémique ou un antécédents de chirurgie du genou. Nous rapportons le cas d'une lésion rare dans la littérature, une rupture bilatérale des tendons rotuliens sans notions de maladies auto-immunes ni de traitement avec des corticostéroïdes. Les lésions bilatérales présentent certaines particularités diagnostiques et thérapeutiques. En effet l'objectivation d'une rotule haute peut être rendu difficile par un aspect controlatéral semblable. Concernant le volet thérapeutique, deux difficultés sont à noter la première réside dans l'absence de référence comparative pour la hauteur patellaire. La deuxième difficulté est l'obligation de différer l'appui à 45 jours. Ce qui est contraignant pour le patient. La technique de laçage décrite par judet couplée à un cadrage provisoire protégeant la suture nous a donné des résultats satisfaisants.

## Introduction

Les lésions du tendon rotulien sont moins communes que celles du tendon quadricipital. Ils surviennent le plus souvent chez un adulte jeune dont l’âge est inferieur à 40 ans suite à un traumatisme direct sur un genou fléchi ou à une flexion contrariée. Le tableau clinique est évident composé d'une douleur associée à une impotence fonctionnelle avec à l'examen une dépression à la palpation du tendon rotulien, une position haute de la rotule et extension active impossible. Les lésions bilatérales des tendons rotuliens sont extrêmement rares et sont souvent associées à une notion de tendinopathie, d'injection de corticoïdes ou de maladies systémiques tels que le lupus érythémateux, l'ostéomalacie ou l'insuffisance rénale chronique. Nous rapportons dans cet article le cas d'une rupture bilatérale des tendons rotuliens chez un patient jeune sans antécédents de tendinopathie ni de maladies systémiques.

## Patient et observation

**Figure 1:**
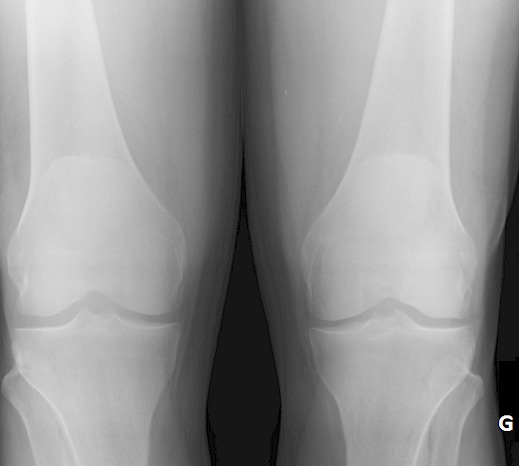
Radiographie de face des deux genoux montrant la position anormalement haute des 2 rotules

**Figure 2:**
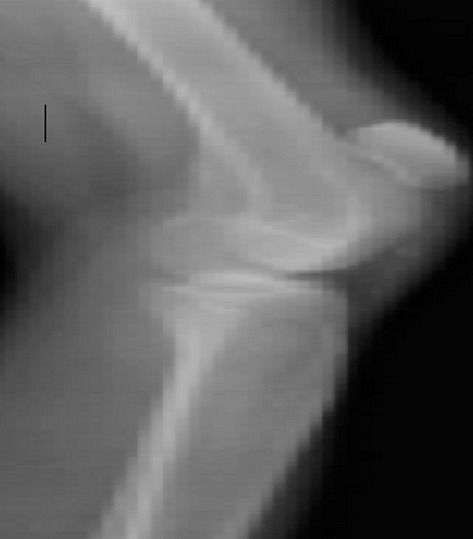
Radiographie de profil du genou droit objectivant la patella alta

**Figure 3:**
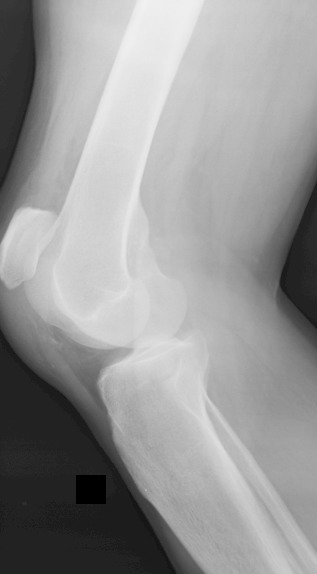
Radiographie de profil du genou gauche objectivant la patella alta

**Figure 4:**
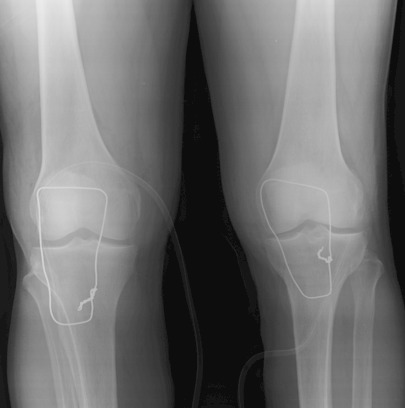
Radiographie de face après réparation du tendon rotulien et protection par cadrage, les deux rotules sont au même niveau

Il s'agit d'un patient de 26 ans sans antécédents notables se présentant aux urgences dans un tableau de douleur intense au niveau des 2 genoux avec impossibilité de la marche suite à un saut de 6 mètres avec réception sur les pieds genoux en flexion. L'examen clinique a objectivé une dépression au niveau des deux tendons rotuliens siégeant pratiquement au même niveau à 3 cm de la pointe de la rotule. La palpation de la rotule montre une position anormalement haute de cette dernière. Le patient présentait également des difficultés d'extension active de la jambe. Les radiographies réalisées aux urgences objectivent une patella alta bilatérale sans fracture associée (Figure 1, Figure 2, Figure 3). Une échographie a été également réalisée et qui a confirmé le diagnostic de rupture bilatérale des deux tendons rotuliens. Le patient a été traité chirurgicalement quatre jours après son traumatisme sous rachianesthésie. Il a été installé en décubitus dorsal avec un garrot pneumatique à la racine du membre. Nous avons pratiqué une incision cutanée verticale antérieure médiane s’étendant de la rotule à la TTA. Après incision verticale de la gaine tendineuse sur sa portion non lésée, le tendon a été libéré sur toute sa longueur et toutes ses faces. Les extrémités du tendon rompu ont été rapprochées et suturées par un laçage selon la technique de Judet [1]. Pour protéger la suture, un cadrage du tendon rotulien avec un gros fil non résorbable passant dans la rotule et la TTA a été mis en place. Une radiographie de contrôle de profil à 30° de flexion comme recommandée par Ait Si Selmi [2] a été réalisée pour régler la hauteur rotulienne selon l'indice de Caton et Descamps (Figure 4). Une immobilisation par attelle amovible a été utilisée pour une durée de 45 jours. L'appui a été différé à J45 vu que la lésion est bilatérale. La rééducation fonctionnelle a débuté précocement au 3^ème^ jour post opératoire en favorisant la flexion passive, limitée à 70° pendant 45 jours. Le patient a été revu régulièrement avec contrôle clinique et radiographique. Après un recul de 6 mois nous avons évalué l’évolution des 2 sutures selon les critères de Siwek [3] se basant sur l’étude de deux éléments: l'amplitude articulaire et la force du quadriceps. Selon ces critères l’évolution Clinique de notre patient a été jugée excellentepour le genou droit alors qu'elle est bonne pour le genou gauche.

## Discussion

La rupture du tendon rotulien est la troisième lésion en terme de fréquence après la rupture du tendon quadricipital et la fracture de la rotule qui cause la rupture de l'appareil extenseur [4]. La lésion la plus fréquente est la désinsertion rotulienne suivie de la rupture en plein corps [5] alors que le mécanisme le plus fréquentest la contraction violente du quadriceps avec un genou fléchi (réception de saut genoux fléchis, relèvement brusque d'une position accroupie) [6]. La rupture bilatérale du tendon rotulien est rare. La plupart des patients rapportent une notion de maladie systémique ou un antécédents de chirurgie du genou [7]. Des signes inflammatoires peuvent êtrenotés sur le siège de rupture en cas de lupus érythémateux [8] ou des dépôts amyloïdes en cas d'insuffisance rénale chronique [9].

Taylor [10] a réparti les ruptures des tendons rotuliens selon leurs causes physiopathologiques en trois groupes: le premier est constitué de ruptures dues a des affections auto-immunes ou systémiques qui provoquent des changements de la structure du tendon rotulien. Le deuxième groupe est composé de ruptures dues à la prise de corticostéroïdes par voie orale ou par voie injectable. Le troisième groupe est fait de ruptures occasionnées par des microtraumatismes répétés. Le cas présenté dans cet article fait partie du troisième groupe, le patient n'a jamais été traité par des corticostéroïdes et n'a pas été pris en charge pour une maladie auto-immune ou de systèmes. Un bilan de maladies de système n'a pas été réalisé comme l'a préconisé Caldas pour tous les patients dont l’âge est supérieur à 40 ans vu que le patient est jeune. Les ruptures bilatérales du tendon rotulien présentent certaines difficultés diagnostiques et thérapeutiques. En effet l'objectivation d'une rotule haute peut être rendu difficile par un aspect controlatéral semblable. De même la difficulté d'extension active de la jambe peut être masquée par des ailerons rotuliens intacts. Les examens radiologiques s'avèrent utiles pour la détection de patella alta. L’échographie et l'IRM peuvent être utilisées en cas de doute diagnostic.

Concernant le volet thérapeutique, deux difficultés sont à noter la première réside dans l'absence de référence comparative pour la hauteur patellaire comme recommandé dans certaines publications [2]. Il est essentiel de placer alors les deux rotules aux mêmes niveaux avec un indice de Caton [11] inférieur à 1,2, des ratios peropératoires sont nécessaires pour assurer le réglage de la hauteur rotulienne. La deuxième difficulté est l'obligation de différer l'appui à 45 jours. Ce qui est contraignant pour le patient. La technique de laçage décrite par judet couplée à un cadrage provisoire protégeant la suture nous a donné des résultats satisfaisants.

## Conclusion

Nous avons rapporté le cas d'une lésion rare dans la littérature, une rupture bilatérale des tendons rotuliens sans notions de maladies auto-immunes ni de traitement avec des corticostéroïdes. Les lésions bilatérales présentent certaines particularités diagnostiques et thérapeutiques. Le cadrage pour protéger la suture est essentiel dans ces cas où les lésions sont de nature dégénératives.
